# Expanding access to HIV services during the COVID-19 pandemic—Nigeria, 2020

**DOI:** 10.1186/s12981-021-00385-5

**Published:** 2021-09-19

**Authors:** Andrew T. Boyd, Ibrahim Jahun, Emilio Dirlikov, Stacie Greby, Solomon Odafe, Alhassan Abdulkadir, Olugbenga Odeyemi, Ibrahim Dalhatu, Obinna Ogbanufe, Andrew Abutu, Olugbenga Asaolu, Moyosola Bamidele, Chibuzor Onyenuobi, Timothy Efuntoye, Johnson O. Fagbamigbe, Uzoma Ene, Ayodele Fagbemi, Nguhemen Tingir, Chidozie Meribe, Adeola Ayo, Orji Bassey, Obinna Nnadozie, Mary Adetinuke Boyd, Dennis Onotu, Jerry Gwamna, McPaul Okoye, William Abrams, Matthias Alagi, Ademola Oladipo, Michelle Williams-Sherlock, Pamela Bachanas, Helen Chun, Deborah Carpenter, David A. Miller, Ugonna Ijeoma, Anuli Nwaohiri, Patrick Dakum, Charles O. Mensah, Ahmad Aliyu, Bolanle Oyeledun, Prosper Okonkwo, John O. Oko, Akudo Ikpeazu, Gambo Aliyu, Tedd Ellerbrock, Mahesh Swaminathan

**Affiliations:** 1 Centers for Disease Control and Prevention, Division of Global HIV and TB, Center for Global Health, 1600 Clifton Road NE, Atlanta, GA 30329 USA; 2 Centers for Disease Control and Prevention Nigeria, Division of Global HIV and TB, Center for Global Health, Abuja, Federal Capital Territory Nigeria; 3 Maryland Global Initiatives Corporation, University of Maryland School of Medicine Nigeria Program, Abuja, Federal Capital Territory Nigeria; 4 Institute of Human Virology (IHVN), Abuja, Federal Capital Territory Nigeria; 5 Center for Integrated Health Program (CIHP), Abuja, Federal Capital Territory Nigeria; 6 AIDS Prevention Initiative Nigeria (APIN), Abuja, Federal Capital Territory Nigeria; 7 Catholic Caritas Foundation Nigeria (CCFN), Abuja, Federal Capital Territory Nigeria; 8 Federal Ministry of Health, Abuja, Nigeria; 9 National Agency for the Control of AIDS, Abuja, Federal Capital Territory Nigeria

**Keywords:** HIV, ART, COVID-19, Nigeria, PEPFAR, CDC

## Abstract

**Background:**

To accelerate progress toward the UNAIDS 90-90-90 targets, US Centers for Disease Control and Prevention Nigeria country office (CDC Nigeria) initiated an Antiretroviral Treatment (ART) Surge in 2019 to identify and link 340,000 people living with HIV/AIDS (PLHIV) to ART. Coronavirus disease 2019 (COVID-19) threatened to interrupt ART Surge progress following the detection of the first case in Nigeria in February 2020. To overcome this disruption, CDC Nigeria designed and implemented adapted ART Surge strategies during February–September 2020.

**Methods:**

Adapted ART Surge strategies focused on continuing expansion of HIV services while mitigating COVID-19 transmission. Key strategies included an intensified focus on community-based, rather than facility-based, HIV case-finding; immediate initiation of newly-diagnosed PLHIV on 3-month ART starter packs (first ART dispense of 3 months of ART); expansion of ART distribution through community refill sites; and broadened access to multi-month dispensing (MMD) (3–6 months ART) among PLHIV established in care. State-level weekly data reporting through an Excel-based dashboard and individual PLHIV-level data from the Nigeria National Data Repository facilitated program monitoring.

**Results:**

During February–September 2020, the reported number of PLHIV initiating ART per month increased from 11,407 to 25,560, with the proportion found in the community increasing from 59 to 75%. The percentage of newly-identified PLHIV initiating ART with a 3-month ART starter pack increased from 60 to 98%. The percentage of on-time ART refill pick-ups increased from 89 to 100%. The percentage of PLHIV established in care receiving at least 3-month MMD increased from 77 to 93%. Among PLHIV initiating ART, 6-month retention increased from 74 to 92%.

**Conclusions:**

A rapid and flexible HIV program response, focused on reducing facility-based interactions while ensuring delivery of lifesaving ART, was critical in overcoming COVID-19-related service disruptions to expand access to HIV services in Nigeria during the first eight months of the pandemic. High retention on ART among PLHIV initiating treatment indicates immediate MMD in this population may be a sustainable practice. HIV program infrastructure can be leveraged and adapted to respond to the COVID-19 pandemic.

## Background

In 2019, Nigeria had an estimated 1.8 million people living with HIV (PLHIV), of whom 1.1 million (61%) were receiving antiretroviral treatment (ART) [1]. A 2018 nationally representative household survey of HIV, the Nigeria HIV/AIDS Indicator and Impact Survey, estimated that only 46.9% of PLHIV aged 15–64 knew their HIV status, while 96.4% of those aware of their status were receiving ART [2]. In April 2019, the US President’s Emergency Plan for AIDS Relief (PEPFAR), which supports the Government of Nigeria in providing HIV services, including technical assistance and direct funding of implementing partners, launched the ART Surge, intended to rapidly increase the proportion of PLHIV identified and linked to treatment, in line with the UNAIDS 90-90-90 HIV targets for epidemic control [3]. As part of the ART Surge, the US Centers for Disease Control and Prevention Nigeria country office (CDC Nigeria), supported by PEPFAR, set a goal to identify, initiate, and retain on ART nearly 340,000 additional PLHIV, focusing efforts in nine states (Benue, Delta, Enugu, Federal Capital Territory, Gombe, Imo, Lagos, Nasarawa, and Rivers) [4].

The coronavirus disease 2019 (COVID-19) epidemic in Nigeria threatened to disrupt the progress of the ART Surge. The Nigeria Federal Ministry of Health confirmed the first COVID-19 case in Nigeria on February 27, 2020, and Nigeria Centre for Disease Control (a domestic agency distinct from CDC Nigeria) activated a national Emergency Operations Center the same day [5]. Additionally, the Nigerian president created a Presidential Task Force to work collaboratively with the Nigeria Centre for Disease Control to manage COVID-19 response resources and coordinate national and state mitigation efforts [6]. Beginning March 31, 2020, and continuing until July 1, 2020, the federal government implemented limitations on interstate travel and urged people to forgo non-essential travel to mitigate COVID-19 transmission [7]. Health facilities continued operations but implemented protocols of distancing in waiting areas and use of personal protective equipment that was not always available. In addition, restrictions on transport made travel to facilities more difficult. Taken together, these mitigation efforts led to both decreased availability and uptake of facility-based health services. To overcome disruption of the ART Surge, CDC Nigeria staff developed and implemented a series of adapted strategies for all PLHIV populations in the nine focus states during February–September 2020. In developing these strategies, we hypothesized that if HIV testing and ART delivery within the ART Surge could be provided more widely in community settings, the number of PLHIV diagnosed and retained in treatment could be maintained or increased. We describe these adapted strategies and their implementation and demonstrate the continued progress of the ART Surge in providing and expanding access to HIV services during the COVID-19 pandemic. Additionally, we describe how virtual community health engagement and health system infrastructure of the ART Surge facilitated COVID-19 mitigation efforts in Nigeria.

## Methods

### Adapted strategies to mitigate COVID-19 transmission

Although COVID-19 was detected in Nigeria in late February 2020, CDC Nigeria, in conjunction with its implementing partners and state-based ART Surge consortia, began developing COVID-19-related adaptations to ART Surge strategies in early February, and had implemented these adaptations by early April. The overarching principle in these adapted strategies was to maintain and expand access to HIV services, including HIV testing, ART initiation, and retention on ART, while mitigating COVID-19 transmission among PLHIV, other health care recipients, and health services staff. In practice, and because facility-based services were more limited, application of this principle meant increasing focus on community-based, rather than facility-based, services, and decreasing the frequency of face-to-face interactions between PLHIV and health staff, both in facility-based and community-based services, without compromising the quality of provided services. In implementing these strategies, we devised corresponding process variables to track their dissemination through time.

Key adapted strategies, and their corresponding process variables, were as follows:

1. Modifying HIV testing to intensify focus on community-based testing. From the start of the ART Surge, a key HIV case-finding strategy was conducting targeted community-based testing.[4, 8] In the COVID-19 context, to maintain HIV case-finding while health facility-based services were more limited, health staff intensified their focus on increasing the proportion of testing done in the community setting. Specifically, dedicated HIV testing and outreach teams entered communities to conduct HIV education, screening, counseling, and testing. These teams focused on higher-prevalence communities as suggested by epidemiologic and programmatic data. Partnerships with community-based organizations facilitated local adaptations to this strategy to ensure outreach team access and effectiveness. [8] The process variables were the number and proportion of PLHIV initiating ART who were diagnosed in the community.
2. Increasing the number of daily doses in ART “starter packs” provided to newly-diagnosed PLHIV from 30 to 90 days. During the ART Surge, prior to COVID-19, newly-diagnosed PLHIV, including those diagnosed in the community, were provided an immediate ART “starter pack” in line with the national Test and Treat strategy.[9] This initial ART dispense, provided in both health facilities and communities, contained 30 daily ART doses. In the context of COVID-19, the number of doses in the ART “starter pack” was increased to 90 daily doses. The process variables were the number and proportion of PLHIV initiating ART with an ART “starter pack” of 90 daily doses.
3. Expansion of ART distribution through community refill sites. During the ART Surge, prior to COVID-19, PLHIV on ART living in rural or poorly-accessible areas could receive ART refills via community drug distribution.[10] In the context of COVID-19, health staff expanded access to ART refill distribution through additional community sites, including in urban and peri-urban settings, providing more PLHIV with more options for picking up needed ART refills. Health staff sent bulk short message service (SMS) generated from electronic medical records (EMRs) for ART refill reminders, including listing of designated nearby community locations for ART refill pickups. At these community sites, staff maintained ART refills for PLHIV to pick up when able, and documented if and when refill pick-ups occurred. The process variables were the number and proportion of PLHIV established in care who received on-time ART refills.
4. Expansion of availability of multi-month dispensing (MMD) of 3–6 months ART for PLHIV established in HIV care. MMD of 3–6 months ART, as opposed to the monthly dispensation of ART, is a key component of PEPFAR-supported HIV services, although in Nigeria, the availability of MMD was limited to PLHIV considered “stable” in care, as indicated by being on ART for at least 6 months and having documented viral load suppression.[11] In the context of COVID-19, criteria for receipt of 3–6 MMD ART were broadened to include those on ART for less than six months and those who were not virally suppressed, enabling more PLHIV established in care to reduce their frequency of ART refill appointments. The process variables were the number and proportion of PLHIV established in care receiving at least 3-month MMD.

In addition to the program process variables described, we examined two program outcome variables key to program growth and quality: the number of PLHIV initiated on ART per month and their retention in HIV care 6 months after initiation.

### Data sources and analysis

As part of ART Surge monitoring and evaluation, both health facilities and community-based HIV service teams reported weekly numbers of newly-diagnosed PLHIV initiating ART in an Excel-based dashboard described elsewhere [4, 8]. Upon implementation of the four key adapted strategies, these teams included data on the corresponding HIV program process variables within the dashboard, including the number of PLHIV initiating ART with an ART “starter pack” of 90 daily doses and the number of PLHIV established in care who received on-time ART refills. In addition, health facility and community-based HIV service teams entered information about newly-diagnosed PLHIV initiating ART into health facility EMRs, and this information was uploaded to the Nigeria National Data Repository (NDR) using Extensible Markup Language (XML) files. The NDR contains a de-identified line-list of all PLHIV in HIV care in the country, permitting real-time data uploads from implementing partners’ health facility EMRs. The NDR also allows retrospective data uploads and corrections, so final numbers of newly-diagnosed PLHIV initiating ART documented in the NDR are frequently greater than the numbers initially reported in the dashboard. The NDR maintains information on the number of ART doses dispensed and timing of dispense in relation to the end of the previous dispense to provide information on individual-level retention on ART, but it does not include information on the additional HIV program process variables in the ART Surge. Thus, the ART Surge weekly dashboard and the NDR act as complementary data sources providing data at different speeds and for different purposes: the weekly dashboard allows rapid program review and introduction of new variables, whereas the NDR is more stable and useful for reviewing overall HIV program review and management, including of PLHIV retention in care.

Using ART Surge dashboard data, we aggregated and examined the reported number of newly-diagnosed PLHIV initiating ART across the nine focus states by month during February–September 2020, and we documented the proportion diagnosed in community settings. Using this time period permitted 6 months of follow-up after ART Surge COVID-19-related adaptations were implemented in April 2020. Also using dashboard data collected during April–September 2020, we examined the number of PLHIV initiating ART each month with an ART “starter pack” of fewer than 90 daily doses or at least 90 daily doses (the equivalent of 3-month MMD). For PLHIV established in care, defined as being a diagnosed PLHIV who had initiated care prior to a given reporting week, we examined the proportion receiving on-time ART refills, defined as receiving an ART dispense on or before the end date of the previous dispense, by month during April–September 2020. This variable was reported in aggregate as the proportion of the number of PLHIV who received a refill over the number of PLHIV expected to receive a refill. Thus, in a given month, the proportion of PLHIV receiving on-time refills could exceed 100%, since PLHIV could pick up a refill prior to when they were due for a refill.

Using NDR data, we examined the ART refill interval for PLHIV established in care by month during February–September 2020 as less than 3-month MMD or at least 3-month MMD. Finally, among monthly cohorts of PLHIV initiating ART during the same time period, we examined their retention in HIV care 6 months after initiation, defined as having received an ART dispense expected to last through the sixth month after initiation, or receiving an ART dispense within 28 days after the drug pickup expected at six months after initiation. Of note, this definition of retention aligns with PEPFAR guidance.[12].

Because this activity consisted of monitoring and improvement of a public health program, using de-identified data and no perceived ethical risk to participants, no informed consent was obtained. This public health program activity review was covered under a non-research determination from the CDC Center for Global Health.

## Results

**Fig. 1:**
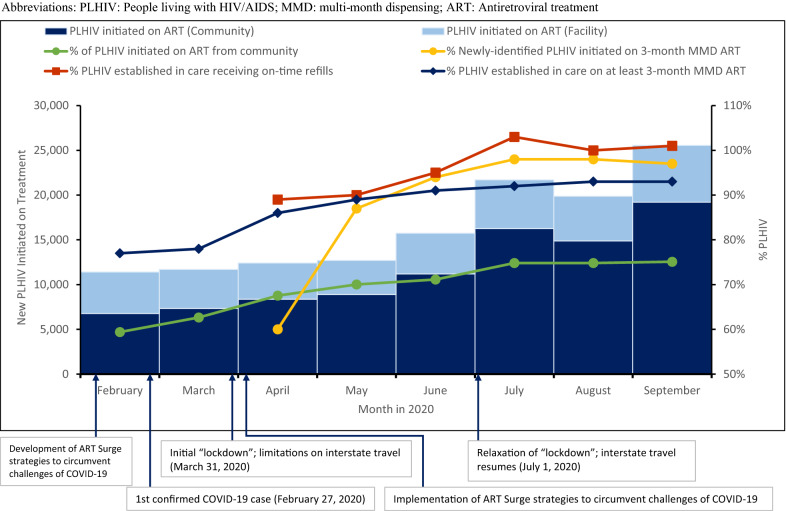
Impact of innovative strategies implemented to circumvent the challenges of COVID-19 during the CDC Nigeria ART Surge, February–September 2020. *PLHIV* People living with HIV/AIDS, *MMD* multi-month dispensing, *ART* Antiretroviral treatment

During February–September 2020, the reported monthly number of PLHIV initiated on ART in the nine focus states was 11,407 in February and increased to a peak of 25,560 in September (Fig. 1). The monthly total number of new ART initiations across April and May was relatively flat, and these achievements coincided in time with introduction and maintenance of COVID-19-related limitations on travel by Nigeria state governments. However, the monthly number of new ART initiations began to increase again in June, even before the limitations were relaxed. The proportion of PLHIV initiating ART who were diagnosed in the community increased throughout the time period, from 59% in February to 75% in September.

The proportion of PLHIV initiated on 3 months of MMD ART by month increased from 60% in April, when the strategy was implemented, to a peak of 98% in August (Fig. 1). This proportion remained 97% through September. Among PLHIV established in care, the proportion of PLHIV receiving on-time ART refills during April–September 2020 increased from 89% to a peak of 103%. The proportion of PLHIV established in care receiving at least 3-month MMD increased from 77% in February to 86% in April, when the strategy of expansion of MMD was implemented, to 93% in September.

Among PLHIV initiating ART documented in the NDR and examined as monthly cohorts during February–September 2020, the proportion retained on ART at 6 months after ART initiation increased from 74% for the February cohort to 92% in the September cohort (Table 1).

**Table 1:** Retention in HIV care six months after ART initiation among PLHIV during the CDC Nigeria ART Surge, by monthly cohort, February–September 2020

Month of ART initiation	February	March	April	May	June	July	August	September
Population of the cohort at end of month of ART initiation	15,002	14,432	14,039	14,895	18,221	24,625	22,364	26,822
Population (6-month retention %) of the cohort after the sixth month post-ART initiation	11,052 (74%)	12,120 (84%)	11,939 (85%)	12,904 (87%)	16,361 (90%)	22,083 (90%)	19,829 (89%)	24,749 (92%)

Retention six months after ART initiation was defined as having received an ART dispense expected to last through sixth month after initiation, or receiving an ART dispense within 28 days after the drug pickup expected at six months after initiation

## Discussion

The COVID-19 pandemic has had a destabilizing effect on HIV epidemic control efforts [13]. A modeling study estimated a 10% increase in HIV deaths over the next 5 years above that expected without the COVID-19 pandemic, likely because of interruptions in ART as HIV services are limited or diverted [14]. HIV service adaptations that ensure continuity of care even during the COVID-19 pandemic are thus essential in health programming to enable favorable clinical outcomes. This need for continuity of HIV services is made clear in a global modeling study that demonstrated that continuing four HIV services, including HIV diagnostic testing, averts at least 100 times more HIV-related deaths than the COVID-19-related deaths attributed to maintaining these services [15]. The ART Surge in Nigeria, perhaps because of its general flexibility of approach to increasing access to HIV services, serves as an example of how HIV services, including community outreach, testing and counseling, and linkage and retention to ART, can be not only maintained but expanded during COVID-19. Within the ART Surge approach, the use of geographically localized incident command structures and consortia with partners allowed rapid development and dissemination of ART service delivery adaptations [4, 8]. When COVID-19 arrived in Nigeria, the staff operating in these structures were able to use this same framework to develop COVID-19 mitigation strategies, incorporate input of technical expertise, and facilitate widespread program implementation of COVID-19-specific adaptations quickly and early in the pandemic. This rapid adaptation was especially true in the key adapted strategy of an intensified focus on community-based case-finding, as evidenced by the increasing proportion of new PLHIV initiating ART who were diagnosed in the community during the examined time period, since this shift simply broadened an original strategy of the ART Surge.

In contrast, the other key adapted strategies represented greater programmatic innovations in service delivery for both PLHIV new on ART and established on ART in Nigeria. In practice, these strategies facilitated more or expanded PLHIV-centered options for ART dispensation. Innovations included providing community ART distribution for both PLHIV initiating ART and expanding ART for PLHIV established in care, and introducing at least 3-month MMD for both PLHIV initiating ART and PLHIV established in care who were not previously eligible. Of note, similar adapted strategies within the COVID-19 pandemic in sub-Saharan African HIV programs have been described [16]. In other settings, PLHIV initating and continuing ART in the community had increased rates of HIV viral suppression compared with those receiving clinic-based care [17], and stable PLHIV established in care who received ART refills provided through community distribution points had equivalent retention in care to those receiving ART in a health facility [18]. Community-based ART initiation is effective in Nigeria [19], and community-based, same-day ART initiation is associated with improved linkage to care and viral suppression, when compared with referral to facility-based initiation [20].

MMD as a differentiated service delivery (DSD) model is widely promoted within PEPFAR-supported HIV programs as a more sustainable approach than traditional monthly clinical visits, but to date MMD has generally been made available to PLHIV considered “stable in care” [21, 22], excluding PLHIV initiating ART or PLHIV established in care who were not virally suppressed. Though the expansion of MMD to these populations may be appropriate in the context of COVID-19, as was implemented in Nigeria, additional data to support maintaining MMD among these populations, including demonstrated retention on ART, after the COVID-19 pandemic has ended are needed. The high 6-month retention of monthly PLHIV cohorts initiating ART in this evaluation indicate that expanded MMD may be a useful service delivery option to achieve positive clinical outcomes among this population and thus may be a sustainable practice. Of note, even as the proportions of PLHIV initiating ART who were retained on ART improved through time, up to 10% were not retained at six months in the later cohorts. This finding suggests that understanding predictors of early interruptions in care and providing interim virtual clinical check-ins and appointment reminders prior to expected ART refill should remain a priority for HIV programs.

The described COVID-19-related adaptations entailed fewer face-to-face clinical interactions, so health staff used virtual interactions to maintain clinical contact with PLHIV. While some PLHIV support activities, specifically tracking and tracing of PLHIV who had interruptions in treatment, were already conducted virtually, COVID-19 obligated the standardization of a programmatic approach to these activities, and these processes remain in place as best practices in the ART Surge. Other activities, like ART adherence counseling, were converted from in-person to virtual activities; a 2015 meta-analysis, including a study from Nigeria, found that technology-supported adherence counseling could achieve desirable clinical outcomes [23, 24]. Of note, certain systems of virtual PLHIV engagement used in the ART Surge, including regular text messages by SMS about HIV services, were also adapted to provide COVID-19 mitigation messaging to the community, an example of using the HIV services infrastructure to support other public health priorities.

While the ART Surge’s wide reach, unique structural components, and flexibility allowed for its continued operations during the COVID-19 pandemic, the ART Surge in turn facilitated COVID-19 surveillance and mitigation efforts in Nigeria. CDC Nigeria and its implementing partners’ staff provided technical input to the national and state-level Emergency Operations Centers on mitigation policies and provided data analysis for situation reports. HIV viral load sample transport networks and instrument platforms were used for COVID-19 testing. Advancing the ART Surge while mitigating the COVID-19 pandemic demonstrates that capacities developed specifically for HIV services have strengthened health systems generally, which can be leveraged to address other pressing public health needs, including future pandemics.

The report has several limitations. The described interventions were of a programmatic design, as opposed to an experimental design, precluding causal inference between the interventions and observed results. Numbers of PLHIV initiating ART reported by HIV service teams in the weekly dashboard were based on self-report from the PLHIV, so duplication of counts of this variable within the weekly dashboard is possible; NDR deduplication algorithms reduce that likelihood in the retention cohort data. Refill data was collected as an aggregate indicator, so individual-level refill timing and interruptions in treatment were not assessed for PLHIV established in care. For analysis of retention, we focused solely on cohorts of PLHIV initiating ART; retention of PLHIV established in care and its relationship with MMD needs further analysis. The ART Surge’s ability to facilitate expansion of community-based services depended on work of implementing partners and community-based organizations, and other national HIV programs without these networks in place may not find the described approach replicable. Although partnerships with community-based organizations facilitated program implementation, formal consultation and evaluation of community perspectives on these described adapted strategies were not conducted. Collecting these perspectives, and using them to ensure services are PLHIV-centered, represent an important next step for the ART Surge. Finally, this report examines aggregate CDC data from nine states and thus may not be representative across all demographic groups, or representative of sites in the nine states outside the CDC-supported site network or in other states.

## Conclusions

A rapid and flexible HIV program response, focused on reducing facility-based and frequent interactions while ensuring delivery of lifesaving ART, was critical in overcoming COVID-19-related service disruptions to the ART Surge. With key COVID-19-specific strategic adaptations in place, CDC Nigeria and its implementing partners were able to expand access to critical HIV services across nine states during the pandemic. High retention on ART among PLHIV initiating treatment indicates immediate MMD in this population may be a sustainable practice. Virtual health system engagement with PLHIV was strengthened. The Nigeria ART Surge infrastructure can be used and adapted to monitor and respond to the COVID-19 pandemic and other public health emergencies, providing an example for how other public health programs could adapt operations in the face of emerging or changing public health program disruptions.

## Data Availability

Reported weekly data are not publicly available because these data are internally-generated program data without the framework for external sharing, but general quarterly PEPFAR programming data, including on numbers of PLHIV on ART, can be found at https://data.pepfar.gov/dashboards Reported Nigeria National Data Repository (NDR) data can be made available upon reasonable request. Data requests can be made directly to the Nigeria Ministry of Health pursuant to the NDR Data Use Agreement. Data requesters can go to https://adb.shieldnigeriaproject.com/index.php/home and register to fill and submit an online data request form, including purpose of analysis and variables requested, along with a concept note or protocol and ethical approval.

## Abbreviations

ART: Antiretroviral Treatment
CDC Nigeria: US Centers for Disease Control and Prevention Nigeria country office
COVID-19: Coronavirus disease 2019
EMR: Electronic medical record
MMD: Multi-month dispensing
NDR: Nigeria National Data Repository
PEPFAR: US President’s Emergency Plan for AIDS Relief
PLHIV: People living with HIV/AIDS
SMS: Short message service
XML: Extensible Markup Language
